# Robust Face Recognition via Multi-Scale Patch-Based Matrix Regression

**DOI:** 10.1371/journal.pone.0159945

**Published:** 2016-08-15

**Authors:** Guangwei Gao, Jian Yang, Xiaoyuan Jing, Pu Huang, Juliang Hua, Dong Yue

**Affiliations:** 1 Institute of Advanced Technology, Nanjing University of Posts and Telecommunications, Nanjing, 210023, China; 2 School of Computer Science and Engineering, Nanjing University of Science and Technology, Nanjing, 210094, China; 3 School of Automation, Nanjing University of Posts and Telecommunications, Nanjing, 210023, China; 4 School of Computer Science and Technology, Nanjing University of Posts and Telecommunications, Nanjing, 210023, China; Jiangnan University, CHINA

## Abstract

In many real-world applications such as smart card solutions, law enforcement, surveillance and access control, the limited training sample size is the most fundamental problem. By making use of the low-rank structural information of the reconstructed error image, the so-called nuclear norm-based matrix regression has been demonstrated to be effective for robust face recognition with continuous occlusions. However, the recognition performance of nuclear norm-based matrix regression degrades greatly in the face of the small sample size problem. An alternative solution to tackle this problem is performing matrix regression on each patch and then integrating the outputs from all patches. However, it is difficult to set an optimal patch size across different databases. To fully utilize the complementary information from different patch scales for the final decision, we propose a multi-scale patch-based matrix regression scheme based on which the ensemble of multi-scale outputs can be achieved optimally. Extensive experiments on benchmark face databases validate the effectiveness and robustness of our method, which outperforms several state-of-the-art patch-based face recognition algorithms.

## Introduction

Object classification is an active topic in the area of pattern recognition [1–8]. Due to the advantages of non-intrusive natural and pronounced uniqueness, face recognition has been an active research topic and has been incorporated into many multimedia applications [9–14], such as surveillance, human machine interaction, access control and photo album management in social networks. Recently, linear regression based face recognition approaches have led to state-of-the-art performance [15–18], with representative examples being sparse representation-based classification (SRC) [15] and linear regression-based classification (LRC) [16]. In SRC, the query sample image is coded as a sparse linear combination of all the training images, and then the classification is made by checking which class yields the least reconstruction error. Many works of SRC have been developed for vision applications, e.g., super-resolution [19, 20], facial expression recognition [21] and human gait recognition [22, 23]. Alternatively, Naseem et al. [16] proposed LRC for face recognition. Based on the assumption that samples from a specific object class lie on a linear subspace, LRC represents a query image as a linear combination of training images of each class. Yang et al. [24] provided an insight into SRC and sought reasonable supports for its effectiveness. They viewed the L_1_-regularizer as having two properties, sparseness and closeness. Sparseness determines a small number of nonzero representation coefficients, and closeness makes the nonzero representation coefficients concentrating on the training samples having the same class label as the test sample. Zhang et al. [18] discussed the working mechanism of SRC and demonstrated that it is collaborative representation rather than L_1_-norm sparseness that improves the classification performance. In their work, a collaborative representation-based classification (CRC) model was presented with a squared L_2_-regularization, which achieves competitive classification performance but with significantly lower complexity than the sparse representation method.

It is worth noting that the majority of studies assume that the testing images are taken under well-controlled settings (e.g., reasonable illumination, poses and variations, without occlusion or disguise). Their performance is degraded when the testing images are contaminated. By introducing an identity matrix *I* as a dictionary to code the outliers (e.g., corrupted or occluded pixels), SRC [15] exhibits excellent robustness and promising performance. However, SRC is not robust to contiguous occlusion such as sunglasses or scarves, as the occlusion level exceeds the breakdown point determined by this algorithm. Yang et al. [25] modified the SRC framework for handling outliers such as occlusions in face recognition by modeling the sparse coding as a sparsity-constrained robust regression problem. He et al. [26] unified the algorithms for error correction and detection by using the additive and multiplicative forms, respectively, and established a half-quadratic framework to solve the robust sparse representation problem. From the viewpoint of dictionary learning, Yang et al. [27] constructed a feature pattern dictionary that captures structured information and prior knowledge of image features to represent the unknown feature pattern weight of a query image. Similarly, Ou et al. [28] developed a clear and noise dictionary simultaneously and applied the learned clear dictionary for classification. Observing the distribution of the reconstruction error image, Yang et al. [29–32] used the nuclear norm to characterize the structural information of an error image and proposed a nuclear norm-based matrix regression model that has achieved state-of-the-art performance for face recognition with occlusion and illumination changes.

In spite of aforementioned tremendous achievements, the small sample size (SSS) problem till remains one of the most fundamental and challenging issues in face recognition community. In many real-world applications such as smart card solutions, law enforcement, surveillance and access control, the available training samples per subject may be very limited [33]. Thus, the performance of these regression-based methods is greatly degraded because the query sample cannot be well represented by the few training samples. To tackle the SSS problem, many efforts have been made in the past few decades. Existing methods mainly fall into three categories. The first are patch-based methods, which generally contain steps of local patch representation, local feature extraction and the combination of classification results [34–36]. However, the patch size has a great impact on the output performance in patch-based methods [37, 38]. The second integrate the local and global features for classification [39, 40] because they can provide complementary information for final results. The third employ different feature extractors to extract multiple types of features, and then utilizes decision level fusion scheme for final classification [41, 42]. We mainly focus on patch-based method in the sequel.

To improve the recognition performance of matrix regression in SSS problem and preserve its outstanding ability dealing with occlusion and illumination changes, in this paper, we propose performing matrix regression on patches. The so-called patch-based matrix regression (PMR) classifies each query matrix patch, and then integrates the recognition outputs of all patches for final decision. Nevertheless, the patch size plays an important role on the final performance in PMR, and the optimal patch size varies greatly across different databases. If the patch size is too small, little information is given, and the method cannot capture the geometric structure of the image; if it is large, the information that can be used is limited. To fully exploit the classification ability and appearance information of different patch sizes, we then devise a multi-scale PMR (MSPMR) scheme by integrating the complementary information from different scales. MSPMR first performs PMR on each scale and then learns optimal scale weights to adaptively fuse multi-scale outputs. To evaluate the performance of the proposed method, we use four databases that involve different recognition tasks: the Extended Yale B, AR and LFW dataset for face recognition without occlusion, the AR database for face recognition with real disguise, and the Extended Yale B dataset for face recognition with block occlusion. The experimental results demonstrate the effectiveness and robustness of the proposed method.

The remainder of the paper is organized as follows. Section 2 briefly reviews two related works. The proposed multi-scale PMR via margin distribution optimization is presented in Section 3. Section 4 conducts extensive experiments, and Section 5 concludes this paper.

## Related Works

### 1. Nuclear norm based matrix regression

By observing the distribution of the reconstruction error image, a nuclear norm-based matrix regression (NMR) [29] model was proposed that uses the nuclear norm to characterize the whole structure of the error image. Here, we define *N*_*i*_ as the number of images from the *i*-*th* class and $N=\sum_{i=1}^{c}N_{i}$ as the total number of training samples from *c* classes. Given a set of *N* training image matrices A_1_, A_2_, …, A_*N*_∈ℜ^*row*×*col*^ and a query image matrix B∈ℜ^*row*×*col*^, the NMR model can be represented as

$$\underset{x}{\text{min}}{\left\|\text{B}-\text{A}(x)\right\|}_{*}+\frac{\lambda }{2}{\left\|x\right\|}_{2}^{2},$$ (1)

where *λ* is the regularization parameter, *x* and A(*x*) = *x*_1_A_1_+*x*_2_A_2_+…+*x*_*N*_A_*N*_ are the representation coefficient vector and the reconstructed image, respectively. Then, the query image can be classified into the class that yields the minimal reconstruction error, i.e.,

$$\text{Identity}(\text{B})=\text{arg }\underset{i}{\text{min}}\left\{r_{i}(\text{B})\right\},$$ (2)

where $r_{i}(\text{B})={\left\|\hat{\text{B}}-{\hat{\text{B}}}_{i}\right\|}_{*}={\left\|\text{A}(x^{*})-\text{A}\left(\delta_{i}(x^{*})\right)\right\|}_{*},$ where x* is the optimal solution of Eq (1) and *δ*_*i*_(*x*) is a vector whose only nonzero entries are the entries in *x* that correspond to Class *i*. We know that NMR is much more robust and effective for face recognition, particularly with respect to occlusion and illumination changes.

### 2. Patch-based CRC

Suppose that we have *c* known pattern classes. Let *X*_*i*_ = [*x*_*i*1_, *x*_*i*2_, …, *x*_*iN*_]$\in {ℜ}^{M\times N_{i}}$ be the matrix formed by the training samples of class *i*, where *N*_*i*_ is the number of training samples of class *i*. Let *X* = [*X*_1_, *X*_2_, …, *X*_*c*_]∈ℜ^*M*×*N*^ be the dataset of all training samples, where $N=\sum_{i=1}^{c}N_{i}$. To alleviate the performance degradation of CRC in the small sample size problem, the patch-based CRC (PCRC) [36] model was proposed. For a given query image *y*, it is first divided into multiple overlapped patches {*y*_1_, *y*_2_,…, *y*_*p*_}. Then, each patch *y*_*i*_ is tackled by representing it as a linear combination over a local dictionary *D*_*i*_. Finally, one can employ the plurality or linear-weighted combination scheme to the recognition outputs for a final decision.

For each patch *y*_*i*_, its representation weights can be obtained by minimizing the following error:

$${\hat{\beta }}_{i}=\underset{\beta_{i}}{\text{arg min}}{\left\|y_{i}-D_{i}\beta_{i}\right\|}_{2}^{2}+\eta {\left\|\beta_{i}\right\|}_{2}^{2}.$$ (3)

Where *D*_*i*_ = [*D*_*i*1_, *D*_*i*2_,…, *D*_*ic*_] denotes the local dictionary located with the same position as *y*_*i*_, *D*_*ik*_ is the sub-dictionary of the *k*-th class. The recognition result of patch *y*_*i*_ is Identity(*y*_*i*_) = argmin_*k*_[43], where $r_{ik}={\left\|y_{i}-D_{ik}{\hat{\beta }}_{ik}\right\|}_{2}/{\left\|{\hat{\beta }}_{ik}\right\|}_{2}$, ${\hat{\beta }}_{ik}$ is the coefficients associated with the *k*-th class.

For clarity, four key components (i.e., muti-scale trick, local patch strategy, structure error and pixel error characterization) of several related methods are compared in Table 1.

**Table 1:** Comparisons of related works.

Method	multi-scale	local patch	structure error	pixel error
CRC[18]				*√*
SRC[15]				*√*
NSC[30]			*√*	
PNN[34]		*√*		
HQ_A[26]				*√*
BlockFLD[38]		*√*		
Volterra[35]		*√*		
PSRC[15]		*√*		*√*
PCRC[36]		*√*		*√*
MSPCRC[36]	*√*	*√*		*√*

## Multi-Scale Patch-Based Matrix Regression (MSPMR)

### 1. Motivation

**Fig 1:**
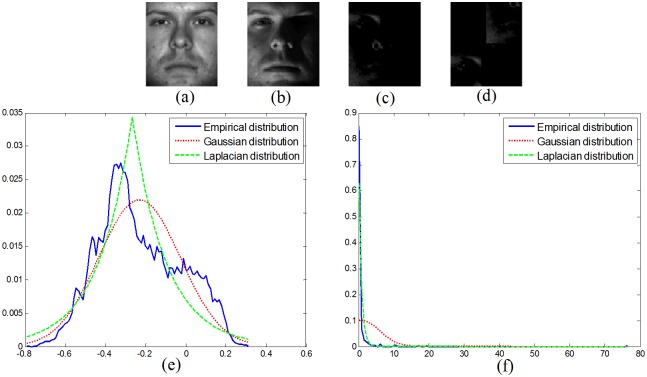
(a) Original image; (b) observed image; (c) error image; (d) rearranged error image; (e) distributions of error image; and (f) distributions of singular values of error image.

In PCRC, each local patch matrix is first converted to a vector, and then the L_2_-norm is used to characterize the reconstruction error. However, the L_2_-norm (or L_1_-norm) is based on pixel values and thus ignores the structural information of the error image. Fig 1 shows an example where the error between (b) and (a) is shown in (c). By re-arranging the pixels of image (c), we can obtain image (d). The following observations can be made:

1. The nuclear norm can better characterize the structure error than the L_1_ or L_2_ norm. For example, the L_2_-norm (or L_1_-norm) value of image (c) is the same as that of image (d), so it is difficult to distinguish between them. Fortunately, the nuclear norm values of images (c) and (d) are 47.75 and 58.14, respectively.
2. From the distribution perspective, we can observe that the distribution of the error image does not follow a Laplacian or Gaussian distribution in Fig 1e. Fortunately, it can be seen from Fig 1f that the singular values of the error image (c) fit the Laplacian distribution well. We know that the nuclear norm is the sum of all singular values of a matrix, which can also be considered as the *l*_1_-norm of the singular value vector. Based on the above example, we believe that the nuclear norm is more suitable to describe the structural error.

### 2. Patch-based matrix regression (PMR)

To make the model robust and efficient for face recognition with occlusion and illumination changes, matrix regression [29, 30, 32] was proposed using the nuclear norm to characterize the structure of the error image. In our patch-based matrix regression, all local patches are denoted in matrix form. Given a set of *N* local patches X_*i*1_, X_*i*2_,…,X_*iN*_∈*R*^*p×q*^ and a query patch Y_*i*_∈*R*^*p×q*^ located at position *i*, Y_*i*_ can be represented linearly using X_*i*1_, X_*i*2_,…,X_*iN*_, i.e.,

$${\text{Y}}_{i}=F(\alpha_{i})+{\text{E}}_{i},$$ (4)

where *F*(*α*_*i*_) = *α*_*i*1_X_*i*1_+*α*_*i*2_X_*i*2_+…+*α*_*iN*_X_*iN*_, *α*_*i*_ = (*α*_*i*1_, …, *α*_*iN*_)^T^ is the representation coefficient vector and E_*i*_ is the representation error. Generally, *α*_*i*_ can be determined by the following regularized model

$${\hat{\alpha }}_{i}=\underset{\alpha_{i}}{\text{arg min}}{\left\|F(\alpha_{i})-{\text{Y}}_{i}\right\|}_{*}+\lambda {\left\|\alpha_{i}\right\|}_{1},$$ (5)

where ||·||_*_ denotes the nuclear norm (the sum of the singular values) on *R*^*p×q*^.

The problem is equivalent to

$$\underset{\alpha_{i},u_{i},E_{i}}{\text{min}}{\left\|{\text{E}}_{i}\;\right\|}_{*}+\lambda {\left\|u_{i}\right\|}_{1},\;s.t.\;F(\alpha_{i})-{\text{Y}}_{i}={\text{E}}_{i}\;,\;\alpha_{i}=u_{i}.$$ (6)

Problem (6) can be solved by the alternating direction method of multipliers (ADMM), which minimizes the following augmented Lagrangian function:

$$\begin{array}{l} L_{\mu }={\left\|{\text{E}}_{i}\;\right\|}_{*}+\lambda {\left\|u_{i}\right\|}_{1}+tr\left(y^{T}\left(\alpha_{i}-u_{i}\right)\right)\;+tr\left(\Lambda^{T}\left(F(\alpha_{i})-{\text{Y}}_{i}-{\text{E}}_{i}\right)\right)\;\\ \;+\frac{\mu }{2}\left({\left\|F(\alpha_{i})-{\text{Y}}_{i}-{\text{E}}_{i}\right\|}_{F}^{2}+{\left\|\alpha_{i}-u_{i}\right\|}_{F}^{2}\right)\;. \end{array}$$ (7)

That is,

$$\begin{array}{l} L_{\mu }={\left\|{\text{E}}_{i}\;\right\|}_{*}+\lambda {\left\|u_{i}\right\|}_{1}+\frac{\mu }{2}\left({\left\|F(\alpha_{i})-{\text{Y}}_{i}-{\text{E}}_{i}+\frac{1}{\mu }\Lambda \right\|}_{F}^{2}+{\left\|\alpha_{i}-u_{i}+\frac{1}{\mu }y\right\|}_{F}^{2}\right)\\ \;-\frac{1}{2\mu }{\left\|\Lambda \right\|}_{F}^{2}-\frac{1}{2\mu }{\left\|y\right\|}_{2}^{2}. \end{array}$$ (8)

The entire algorithm is briefly summarized in **Algorithm 1**, which mainly consists of two steps: a soft-thresholding operator [44] and a singular value thresholding operator [45].

Based on the optimal solution *α*_i_*, we can obtain the reconstructed image of Y as ${\hat{\text{Y}}}_{i}=F(\alpha_{i}^{*})$. Let *δ*_*k*_: *R*^*n*^→*R*^*n*^ be the characteristic function that selects the coefficients associated with the *k*-th class. For *α*∈*R*^*n*^, *δ*_*k*_(*α*) is a vector whose nonzero entries are the entries in *α* that are associated with Class *k*. Using the coefficients associated with the *k*-th class, one can obtain the reconstruction of Y_*i*_ in Class *k* as ${\hat{\text{Y}}}_{ik}=F\left(\delta_{k}(\alpha_{i}^{*})\right)$.

**Algorithm 1.** Solving problem (6) via ADMM

**Input:** A set of *N* patches X_*i*1_, X_*i*2_,…,X_*iN*_∈*R*^*p×q*^ and a query patch Y_*i*_∈*R*^*p×q*^, parameters *λ* and *μ*, the termination condition parameter *ε*.

1: Fix the others and update $u_{i}^{k+1}$ by

$$u_{i}^{k+1}=\underset{u_{i}}{\text{arg min}}\frac{\lambda }{\mu }{\left\|u_{i}\right\|}_{1}+\frac{1}{2}{\left\|u_{i}-\left(\alpha_{i}{}^{k}+\frac{1}{\mu }y^{k}\right)\right\|}_{F}^{2};$$

2: Fix the others and update $\alpha_{i}^{k+1}$ by

$$\alpha_{i}^{k+1}=\underset{\alpha_{i}}{\text{arg min}}\frac{\mu }{2}\left({\left\|\alpha_{i}-u_{i}^{k+1}+\frac{1}{\mu }y^{k}\right\|}_{F}^{2}+{\left\|F(\alpha_{i})-{\text{Y}}_{i}-{\text{E}}_{i}{}^{k}+\frac{1}{\mu }\Lambda^{k}\right\|}_{F}^{2}\right);$$

3: Fix the others and update ${\text{E}}_{i}^{k+1}$ by

$${\text{E}}_{i}^{k+1}=\underset{{\text{E}}_{i}}{\text{arg min}}\;\frac{1}{\mu }{\left\|{\text{E}}_{i}\right\|}_{*}+\frac{1}{2}{\left\|{\text{E}}_{i}-\left(F(\alpha_{i}^{k+1})-{\text{Y}}_{i}+\frac{1}{\mu }\Lambda^{k}\right)\right\|}_{F}^{2};$$

4: Update the multipliers

$$y^{k+1}=y^{k}+\mu \left(\alpha_{i}^{k+1}-u_{i}^{k+1`}\right),\;\Lambda^{k+1}=\Lambda^{k}+\mu \left(F(\alpha_{i}^{k+1})-{\text{Y}}_{i}-{\text{E}}_{i}^{k+1}\right);$$

5. If the termination condition is satisfied, go to 6; otherwise go to 1.

6. **Output:** Optimal coding vector *x*^*k*+1^.

The corresponding class reconstruction error is defined as

$$e_{ik}({\text{Y}}_{i})={\left\|{\hat{\text{Y}}}_{i}-{\hat{\text{Y}}}_{ik}\right\|}_{*}={\left\|F(\alpha_{i}^{*})-F\left(\delta_{k}(\alpha_{i}^{*})\right)\right\|}_{*}.$$ (9)

The recognition output *z*_*j*_ of query patch Y_*i*_ is denoted as Identity (Y_*i*_) = argmin_*k*_{*e*_*ik*_(Y_*i*_)}.

**Fig 2:**
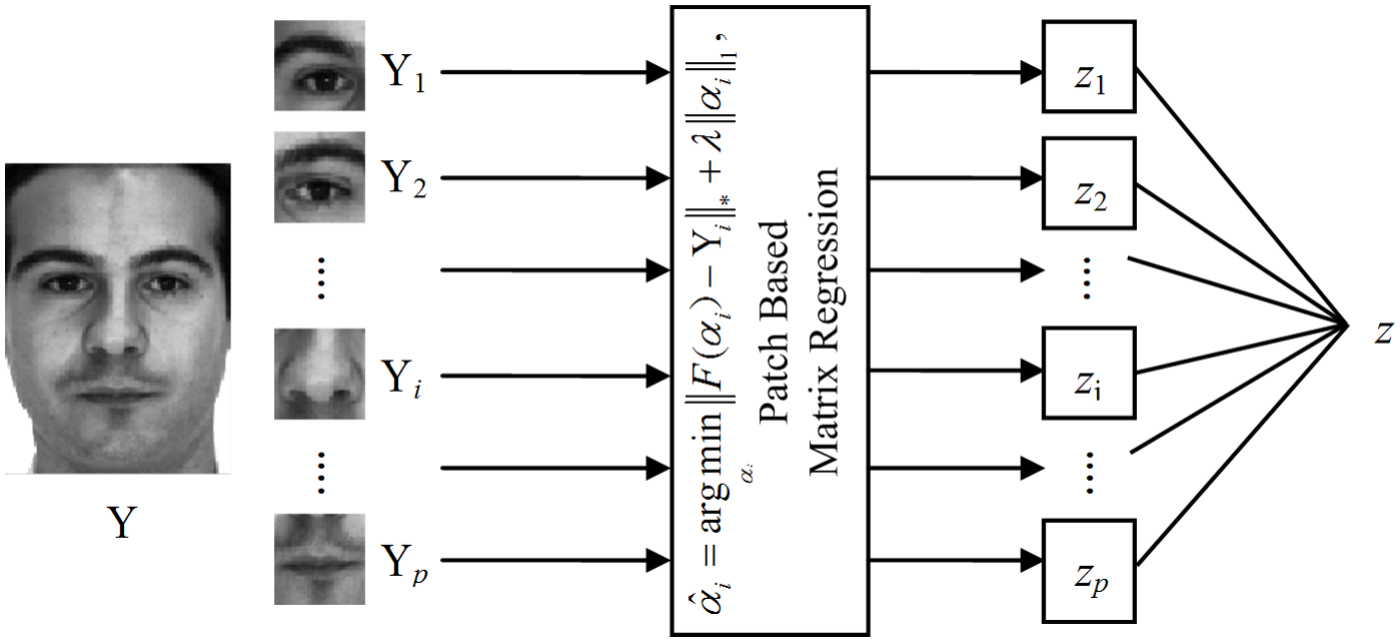
Diagram of patch-based matrix regression for face recognition.

Then we can combine the classification outputs of all patches by linear weighted combination [37], probabilistic model [40], kernel plurality [34] or majority voting [35]. In this paper, we use majority voting in the final decision making. Fig 2 shows the main diagram of the patch-based matrix regression for face recognition.

### 3. Multi-scale ensemble

**Fig 3:**
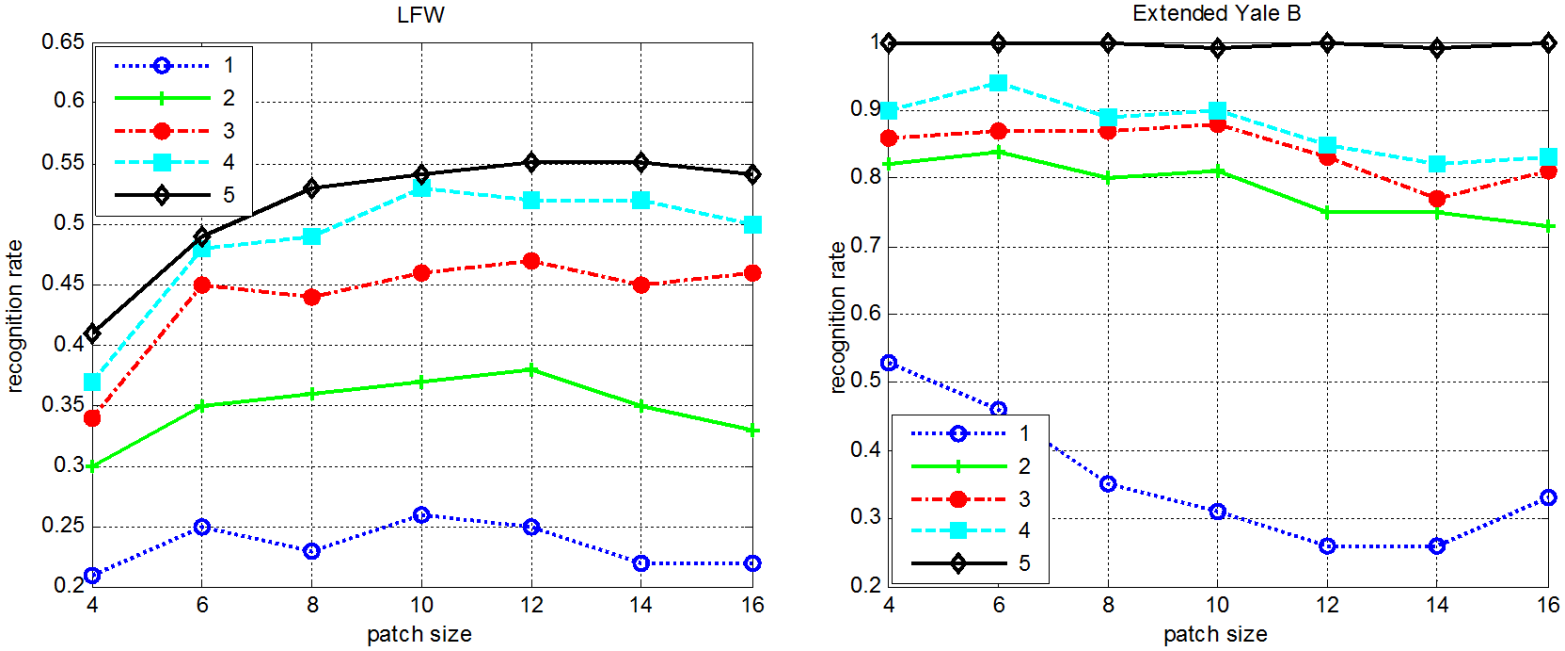
Impact of patch size on PMR (1–5 denote the training sample size per subject).

From the previous introduction of PMR, we can find that the patch size plays an important role on the final output performance. In addition, how to set an optimal scale in advance for various databases remains unclear. Fig 3 exhibits the recognition rates curves versus different training sample sizes and patch sizes on the LFW and Extended Yale B databases, respectively. From Fig 3, the following observations can be made. First, the optimal patch size varies greatly between different databases. Second, the optimal patch size also varies a lot under the variation of the training sample size per person. To tackle the aforementioned difficulties, the recognition outputs of multi-scale PMR can be fused optimally; thus, the complementary information from different scales can be fully applied to further enhance the recognition performance. Motivated by [36], we also incorporate the ensemble learning scheme into our method to integrate multi-scale outputs.

**Fig 4:**
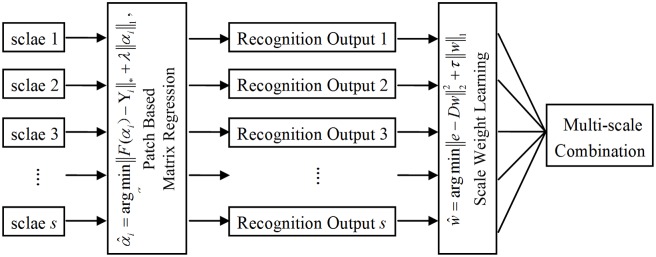
Flow chart of multi-scale learning for PMR.

The diagram of the proposed multi-scale PMR is shown in Fig 4. In the following text, we first formulate the multi-scale ensemble problem, and then introduce a margin distribution optimization to obtain the optimal solution.

#### Problem formulation

Suppose that we have two scales and two sample classes labeled +1 and -1. For any query sample, we can obtain its classification result +1 or -1 on each scale. Thus, each sample will have four possible classification results on these two scales, such as {-1,+1}, {+1,+1}, {-1,-1} and {+1, -1}. Given an available training data set, our goal is to learn a classification function *f*, which can make it possible to classify all the given samples exactly.

Given a sample set *S* = {(*x*_*i*_,*z*_*i*_)} (*i* = 1, 2, …, *n*, *z*_*i*_ is the label of *x*_*i*_) and *s* scales, the classification results of samples *x*_*i*_ on these *s* scales can form a space *H*∈*R*^*n×s*^. Denote by *w* = [*w*_1_, *w*_2_, …, *w*_*s*_] the scale fusion vector, and $\sum_{j=1}^{s}w_{j}=1$.

**Definition 1** [36]: For multi-class classification tasks, the classification results of a given query sample *x*_*i*_∈*S* on these *s* different scales are denoted as [4], *j* = 1, 2, …, *s*. Then the decision matrix *D* = {*d*_*ij*_}, *i* = 1, 2, …, *n*, *j* = 1, 2, …, *s* can be defined as

$$d_{ij}=f(z_{i},h_{ij})=\{\begin{array}{c} +1,if\;z_{i}=h_{ij}\\ -1,if\;z_{i}\neq h_{ij} \end{array},$$ (10)

where *z*_*i*_ is the label of the sample *x*_*i*_.

**Definition 2** [36]: For a query sample *x*_*i*_∈*S*, [4] (*j* = 1, 2, …, *s*) are the classification results on these *s* different scales. Then the ensemble margin of *x*_*i*_ is denoted as

$$\varepsilon (x_{i})=\sum_{j=1}^{s}w_{j}d_{ij}.$$ (11)

The ensemble loss of *x*_*i*_ can be denoted as [36]

$$l_{x_{i}}=l(\varepsilon (x_{i}))=l\left(\sum_{j=1}^{s}w_{j}d_{ij}\right),$$ (12)

Where *ε*(*x*_*i*_) is the ensemble margin of sample *x*_*i*_.The square loss applied in CRC [18], SRC [15] and least square regression [16] can be used here to evaluate the ensemble loss. For a sample set *S*, its ensemble square loss can be formulated as

$$\begin{array}{ll} l(S) & =\sum_{i=1}^{n}l_{x_{i}}=\sum_{i=1}^{n}{\left[1-\varepsilon (x_{i})\right]}^{2}\\ & =\sum_{i=1}^{n}{\left[1-\sum_{j=1}^{s}w_{j}d_{ij}\right]}^{2}={\left\|e_{1}-Dw\right\|}_{2}^{2}, \end{array}$$ (13)

where *e*_1_ is a column vector whose entries are 1.

#### Algorithm of MSPMR

In order to obtain the optimal scale fusion weights, the ensemble square loss in Eq (13) should be minimized. Nevertheless, the solutions may be non-unique for this linear system. Intuitively, we should impose constraint on the objective function in Eq (13) to make the solution unique and stable. Also, Shawe-Taylor [46] has provided the bound on the generalization error and pointed out that both the norm of *w* and the ensemble square loss should be optimized simultaneously to enhance the generalization ability.

As in [36], the following constrained *l*_1_-regularized least square optimization can be used to obtain the optimal scale weights [47]:

$$\begin{array}{l} \hat{w}=\underset{w}{\text{arg min}}{\left\|e-Dw\right\|}_{2}^{2}+\tau {\left\|w\right\|}_{1}\\ \;s.t.\;\sum_{j=1}^{s}w_{j}=1,w_{j}>0 \end{array}$$ (14)

where *τ* is a regularization parameter and the regularization term can help to achieve a stable solution. Constraint $\sum_{j=1}^{s}w_{j}=1$ can be converted to *e*_2_*w* = 1, where *e*_2_ = [1,1, …,1] is a row vector whose length is *s*. Then, we have

$${\left\|e-Dw\right\|}_{2}^{2}={\left\|e-Dw+1-e_{2}w\right\|}_{2}^{2}={\left\|[e;1]-[D;e_{2}]w\right\|}_{2}^{2}$$ (15)

Denoted by $\hat{e}=[e;1]$ and $\hat{D}=[D;e_{2}]$, then we have [36]

$$\hat{w}=\underset{w}{\text{arg min}}{\left\|\hat{e}-\hat{D}w\right\|}_{2}^{2}+\tau {\left\|w\right\|}_{1}\;s.t.\;w_{j}>0,j=1,2,\ldots ,s$$ (16)

**Algorithm 2.** Algorithm of multi-scale ensemble learning for PMR

1: Choose *s* patch scale *δ* = [*δ*_1_, *δ*_2_, …, *δ*_*s*_];

2: Obtain the recognition outputs [4] by PMR;

3: Obtain the decision matrix

$$d_{ij}=f(z_{i},h_{ij})=\{\begin{array}{c} +1,if\;z_{i}=h_{ij}\\ -1,if\;z_{i}\neq h_{ij} \end{array};$$

4: Learn the fusion weights

$$\hat{w}=\underset{w}{\text{arg min}}{\left\|\hat{e}-\hat{D}w\right\|}_{2}^{2}+\tau {\left\|w\right\|}_{1}\;s.t.\;w_{j}>0,j=1,2,\ldots ,s.$$

Duo to the fact the decision matrix is usually very small, the scale fusion weights *w* can be simply obtained by some commonly used *l*_1_-minimization solvers. In our method, *l*_1__*ls* [48] is employed. Based on the above description, the proposed multi-scale PMR (MSPMR) scheme is summarized in **Algorithm 2**. Once the optimal scale fusion weights are achieved, the recognition output for an arbitrary sample *x*_*i*_ can be represented as $z_{i}=\underset{k}{\text{arg max}}\left\{\sum w_{j}|h_{ij}=k\right\}$.

### 4. Computational complexity

In this subsection, we will evaluate the computational complexity of the proposed method. Since the multi-scale fusion weights can be learned off-line, we only discuss the computational complexity of the on-line recognition process involved in the proposed method. As illustrated in Algorithm 2, the proposed face recognition method takes major cost on patch-based matrix regression process. We observe that there are four factors affecting the cost in our method: the training sample size *N*, the dimension of one patch *m* = *p×q*, the number of iterations *k* in Algorithm 1, the patch number in one image *M*.

As described in [30], the matrix regression of each patch takes *O*(*k*(*m*^1.5^+*mN*+*N*^2^)) (in the case that *p* = *q*) cost. For *M* image patch, the computational cost is *O*(*k*(*m*^1.5^+*mN*+*N*^2^)*M*). In addition, the scale number *s* also affect the final running time. Therefore, the computational cost of the proposed method is about *O*(*sk*(*m*^1.5^+*mN*+*N*^2^)*M*). In Section 4, we will further compare the proposed method with the state-of-the-art approaches in terms of CPU runtime.

## Experimental Results and Discussion

In this section, we conduct experiments on the benchmark face databases and the proposed method is compared with state-of-the-art models. For each method, we perform 20 runs of test on each database, and the average recognition rates and the corresponding standard deviations are reported. As in [36], seven scales are adopted in our MSPMR, and the patch sizes are 4×4, 6×6, 8×8, 10×10, 12×12, 14×14 and 16×16. In single scale-based PNN, PSRC, PCRC and PMR, the patch size is 10×10, and the patches overlap with their neighbors by 5 pixels. For PMR and MSPMR, we choose the optimal *λ*∈[0.01,0.1]. Parameter *τ* is set as 0.1 for MSPMR. It should be mentioned here that all experiments are done on the original face images, without any feature extraction or image preprocessing step. Some face image datasets were used in this paper to verify the performance of our methods. These face image datasets are publicly available for face recognition research, and the consent was not needed. The face images and the experimental results are reported in this paper without any commercial purpose.

As in [36], to learn the optimal scale weights, the training set is divided into subset1 (one image per person) and subset2 (the reminder of the training set). Then, PMR is used to classify samples from subset1 utilizing subset2 as the gallery set and the optimal weights on the seven scales can be learned. It should be noted that at least two samples per person are required to find the optimal scale fusion weights.

### 1. Face recognition without occlusion

In this subsection, we test the MSPMR for face recognition without occlusion on four face databases (Extended Yale B [49] and AR [50] in controlled environments together with the LFW [36] in uncontrolled environments). The baseline CRC [18], SRC [15], NSC [30], and patch-based methods including PNN [34], BlockFLD [38], Volterra [35], PCRC and MSPCRC [36] are used for comparison.

#### Extended Yale B database

**Fig 5:**
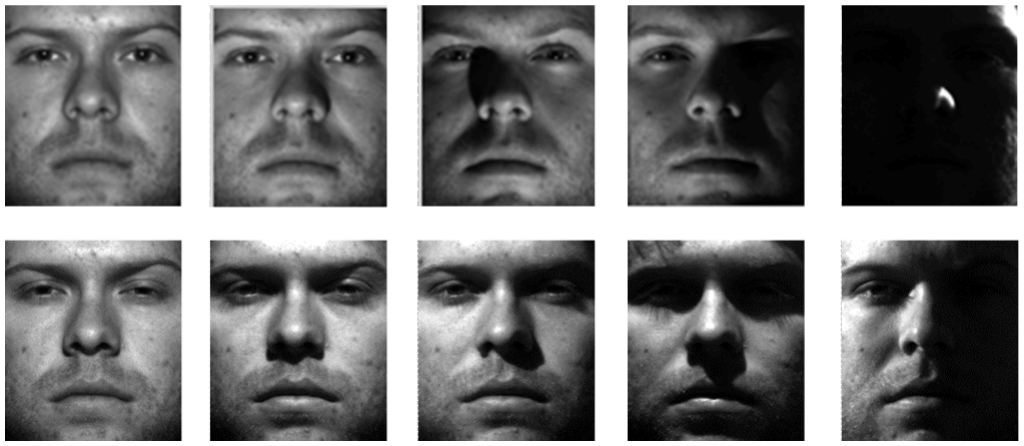
Sample images of a person under various illumination conditions in the Extended Yale B database from different sessions.

The first experiment was conducted on the Extended Yale B database; it includes 38 human objects in 9 poses under 64 illumination changes [49]. 64 images of a person with a particular pose are acquired at a camera frame rate of 30 frames per second, so the variations in the head pose and facial expression are small. All the frontal images marked with P00 are utilized in this experiment, and each is rehsaped to 32×32. Some examples are shown in Fig 5. For each subject, 2~5 samples are randomly selected from the first 32 images for training, and another 5 samples are randomly chosen from the rest 32 images for testing. Table 2 tabulates the experimental results.

**Table 2:** Recognition rates (%) on the Extended Yale B database.

Method	2	3	4	5
CRC[18]	61.3±16.6	74.0±15.5	81.4±17.6	87.8±13.7
SRC[15]	64.2±17.2	74.2±15.2	82.6±16.8	89.0±12.5
NSC[30]	72.1±15.6	79.4±14.5	87.7±14.0	92.7±11.3
PNN[34]	60.8±14.4	65.6±15.1	73.8±15.8	79.7±14.6
BlockFLD[38]	79.5±8.4	83.8±7.8	88.3±5.4	90.7±5.5
Volterra[35]	69.8±12.9	79.5±12.3	84.0±14.0	86.4±9.6
PCRC[36]	75.7±12.6	82.8±12.4	88.7±8.4	92.0±8.2
MSPCRC[36]	83.0±9.2	88.4±10.1	92.5±6.8	95.0±6.6
PMR	79.4±12.4	84.9±14.1	90.7±10.9	95.0±9.5
MSPMR	**85.0±11.9**	**90.0±12.5**	**95.0±9.4**	**97.0±8.6**

It can easily be seen that MSPMR obtains the best recognition performance for all tests. Compared with PCRC and MSPCRC, PMR and MSPMR lead to much better results, thus verifying the effectiveness of characterizing the reconstruction error by the nuclear norm.

#### AR database

**Fig 6:**
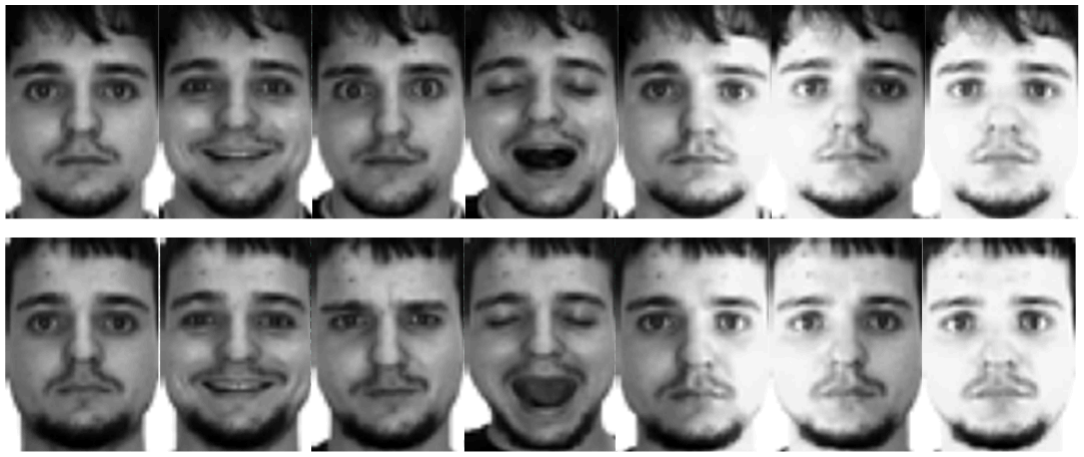
Sample images of one person from the AR database.

The AR database [50] gathers over 4,000 color face images from 126 subjects, containing frontal facial images with different lighting conditions, facial expressions and occlusions. Pictures of 120 subjects were taken in two sessions (separated by two weeks), and each has 13 color images. As in [18], in this experiments, we choose a subset with only illumination and expression changes, which includes 50 male objects and 50 female objects. Fourteen face images (seven from each session) of these 100 individuals are selected and used. For each object, 2~5 samples from session 1 are randomly chosen for training, and another 3 samples from session 2 are randomly chosen for testing. All the images are manually cropped and then resized to 32×32 pixels. Some sample images of one person are presented in Fig 6.

The recognition results of different methods are listed in Table 3. The proposed methods always achieve better performance than the other methods. We can observe that in AR database, multi-scale ensemble learning in MSPMR leads to limited improvement over PMR. As described in [36], the reason may be that in this database, the average weight value for the scale 10×10 is approximately 0.9, indicating that patch size 10×10 is a proper choice for PMR in the AR database.

**Table 3:** Recognition rates (%) on the AR database.

Method	2	3	4	5
CRC[18]	69.9±12.6	80.6±10.4	83.8±9.6	89.1±6.2
SRC[15]	69.7±14.8	79.0±10.6	83.5±8.9	88.2±5.7
NSC[30]	77.6±8.4	84.4±6.7	87.8±5.3	91.1±3.9
PNN[34]	72.7±14.2	82.4±9.3	87.6±8.0	92.2±6.0
BlockFLD[38]	71.5±11.5	78.6±9.8	84.2±8.7	87.6±4.2
Volterra[35]	65.4±12.0	74.9±11.1	79.8±10.5	85.2±6.8
PCRC[36]	82.2±11.3	87.7±9.4	89.9±8.5	92.9±6.7
MSPCRC[36]	82.3±11.5	87.8±10.5	90.2±9.1	93.6±7.6
PMR	83.9±11.2	90.6±7.9	93.2±7.1	95.6±5.8
MSPMR	**87.3±9.0**	**91.5±7.6**	**93.9±7.2**	**96.6±5.8**

#### LFW database

**Fig 7:**
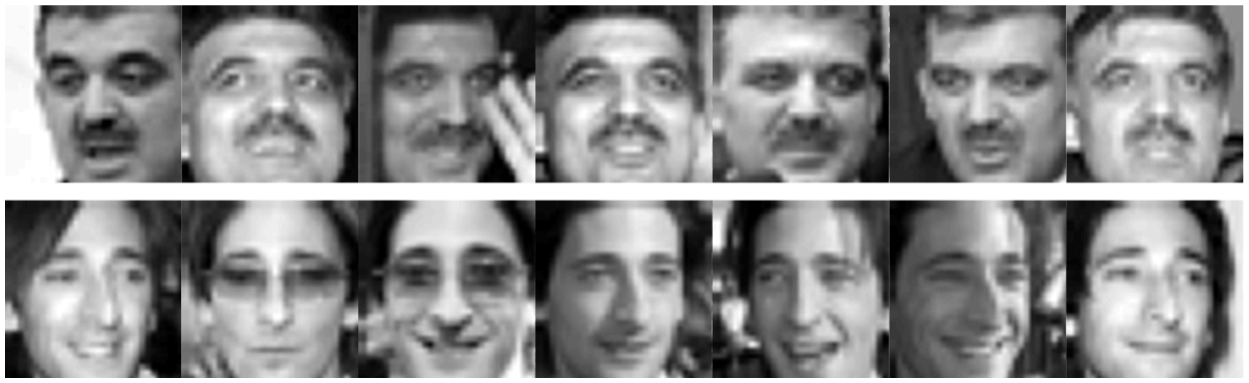
Sample images of the LFW database.

Labeled Faces in the Wild (LFW) [43] is a large-scale database of face photographs designed for unconstrained face recognition with variations in pose, illumination, expression, misalignment and occlusion; it contains images of 5,749 subjects. LFW-a is an extension of LFW after a commercial face alignment software is applied. As in [36], the objects who have more than ten samples are gathered to form a dataset with 158 objects from LFW-a. All the images are manually cropped and then resized to 32×32 pixels. Fig 7 shows some sample images from this database. For each subject, we randomly choose 2~5 samples for training and another 2 samples for testing.

Table 4 shows the face recognition of each method on the LFW dataset. From Table 4, we can clearly see that the performance of our PMR and MSPMR are superior to that of all other methods. Meanwhile, the recognition performance is greatly improved by MSPMR.

**Table 4:** Recognition rates (%) on the LFW database.

Method	2	3	4	5
CRC[18]	24.7±2.1	31.9±2.4	37.8±2.6	42.0±3.2
SRC[15]	24.4±2.4	32.7±3.2	38.7±2.4	44.1±2.6
NSC[30]	26.6±2.2	33.7±2.5	40.6±2.1	45.2±2.7
PNN[34]	23.1±2.4	28.1±3.1	33.2±3.1	37.4±2.7
BlockFLD[38]	18.0±2.1	22.3±2.1	26.2±2.6	28.4±2.5
Volterra[35]	26.0±3.0	32.0±3.4	36.4±3.3	40.3±2.7
PCRC[36]	32.0±1.9	37.0±2.8	40.2±2.5	42.9±2.6
MSPCRC[36]	35.0±1.6	41.1±2.8	46.0±3.0	49.0±2.9
PMR	37.3±2.0	45.1±2.6	50.7±2.7	55.6±2.4
MSPMR	**40.0±2.5**	**50.0±2.4**	**54.2±3.6**	**58.2±3.3**

### 2. Face recognition with occlusion

In the following experiments, we evaluate the robustness and effectiveness of the proposed method when face images encounter with different occlusions, like real disguise or block occlusion. In this subsection, our method is compared with CRC [18], SRC [15], NSC [30], HQ_A and HO_M [26], PSRC [15], PCRC and MSPCRC [36].

#### Face recognition with real disguise

**Fig 8:**
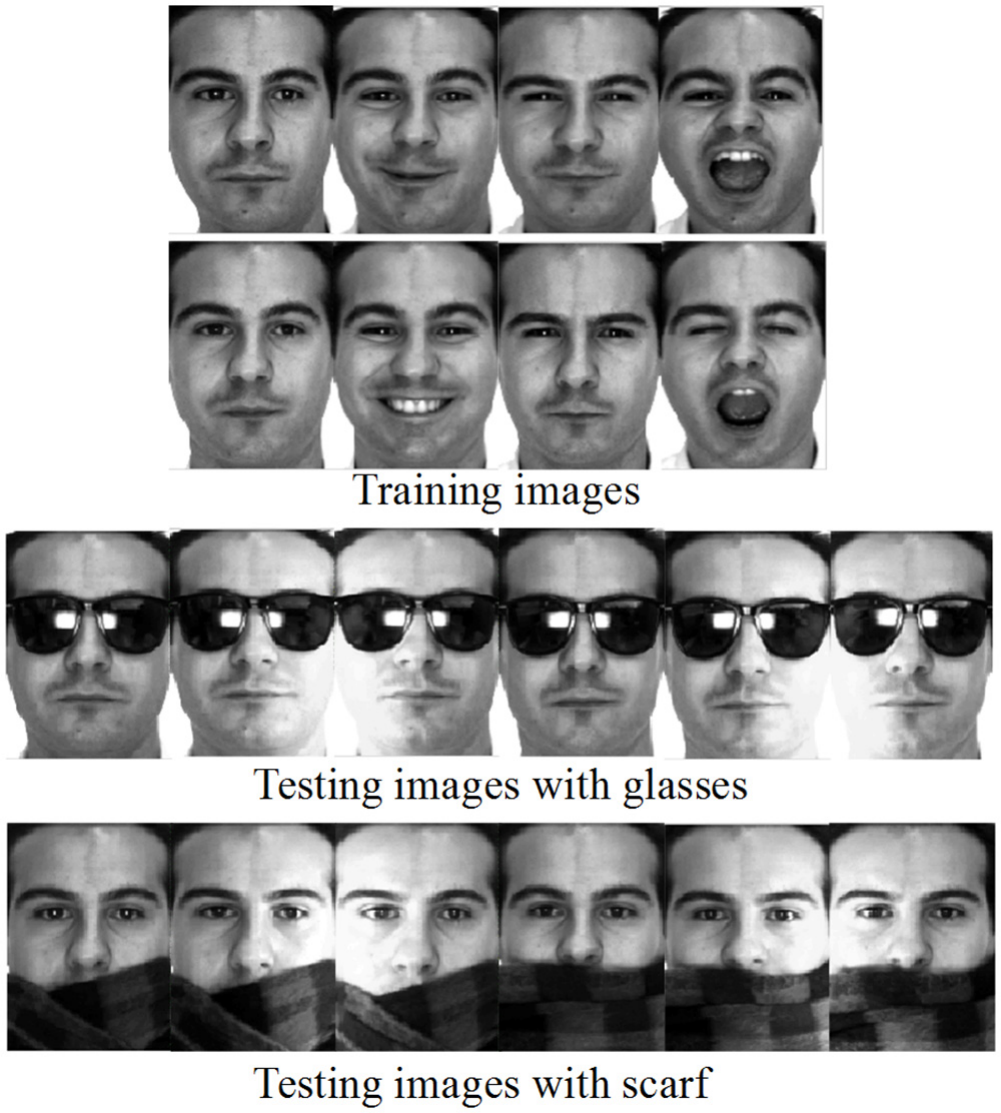
Training and testing images of a person in the AR database.

As in [29, 32], a subset of the AR face database is applied, containing 50 males and 50 females. Each face image is manually cropped and normalized to a size of 42×30. Fig 8 shows the sample images for one person from the AR database. In our experiment, for each individual, the first four images (with various facial expressions) from session 1 and session 2 are chosen to form the training set. Two image sets with sunglasses and scarves are used for testing, each of which includes 600 images (three images per session of each individual). For each individual, 2~5 samples are randomly chosen from the training set and another 3 samples from the testing set to evaluate the performance of each method.

The recognition results of each method are shown in Tables 5 and 6, from which we can see that the patch-based method achieves better performance than the corresponding original holistic ones. PMR also gives better results than PCRC or MSPCRC. MSPMR obtains the best performance among all the competing methods when testing images with sunglasses and achieves comparable results when testing images with scarves.

**Table 5:** Recognition rates (%) on the AR database testing with sunglasses.

Method	2	3	4	5
CRC[18]	23.6±8.3	29.4±7.2	35.4±8.0	40.4±7.8
SRC[15]	30.7±8.0	36.0±7.4	39.7±8.3	41.0±7.9
NSC[30]	58.8±12.9	68.8±9.8	75.2±7.6	77.8±6.3
HQ_A[26]	54.5±10.0	62.0±9.4	67.2±10.5	68.7±9.8
HQ_M[26]	55.7±10.4	63.4±9.7	67.7±10.4	70.8±10.0
PSRC[15]	54.6±14.1	67.2±10.9	74.2±10.9	78.8±8.5
PCRC[36]	63.4±14.2	69.2±11.6	72.8±11.0	74.6±8.7
MSPCRC[36]	68.9±16.6	72.5±13.5	75.1±11.7	76.5±8.5
PMR	65.4±14.7	75.9±10.2	83.6±7.8	86.8±5.5
MSPMR	**76.6±10.8**	**85.6±7.0**	**88.9±5.8**	**91.1±5.5**

**Table 6:** Recognition rates (%) on the AR database testing with scarves.

Method	2	3	4	5
CRC[18]	31.2±7.4	43.1±9.5	49.6±9.9	53.6±9.3
SRC[15]	29.6±7.3	41.4±8.9	47.4±8.5	52.6±5.5
NSC[30]	45.5±8.9	55.1±7.2	59.8±7.0	63.5±5.5
HQ_A[26]	39.8±7.8	49.0±7.7	55.3±8.1	59.4±7.6
HQ_M[26]	39.4±8.0	48.7±8.3	54.4±8.6	58.6±7.5
PSRC[15]	73.3±9.3	82.3±5.5	86.7±4.5	89.3±3.8
PCRC[36]	75.5±10.3	82.8±5.6	86.2±4.5	87.7±3.5
MSPCRC[36]	77.7±12.4	84.7±5.7	87.8±4.9	89.6±4.6
PMR	82.5±8.7	90.2±3.7	92.4±2.1	93.9±1.6
MSPMR	**84.0±8.6**	**90.9±4.1**	**93.9±2.5**	**94.9±3.2**

#### Face recognition with block occlusions

**Fig 9:**
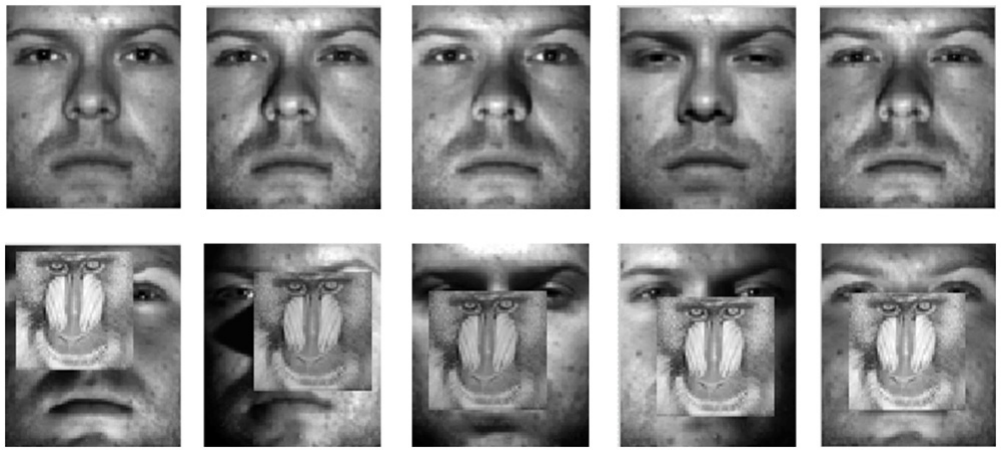
Training (the first row) and testing (the second row) sample images of a person in the Extended Yale B database.

In this subsection, we evaluate the robustness of our method against block occlusions. We adopt Subsets 1 and 2 of the Extended Yale B database for training and Subset 3 for testing. All the face images are normalized to 48×42 pixels. The testing images are corrupted by a randomly located square block of a “baboon” image with an occlusion level of 40%. Fig 9 shows the training and testing sample images for one person from the Extended Yale B database. For each individual, 2~5 samples are randomly chosen from the training set and another 5 samples from the testing set to evaluate the performance of each method.

The face recognition results of each method are tabulated in Table 7. We can see that by characterizing the reconstruction error with the nuclear norm, NSC overall outperforms CRC, SRC, HQ_A and HQ_M. By virtue of the patch trick, our PMR always outperforms PCRC and PSRC. By incorperating the multi-scale ensemble learning trick, the proposed MSPMR achieves the best performance among all the competitive methods.

**Table 7:** Recognition rates (%) on the Extended Yale B database with block occlusion.

Method	2	3	4	5
CRC[18]	40.4±12.5	52.3±12.8	61.1±11.2	67.4±8.9
SRC[15]	50.8±11.5	62.3±10.0	67.5±13.0	71.2±14.5
NSC[30]	67.2±9.5	76.0±8.0	82.6±8.0	85.2±8.7
HQ_A[26]	51.6±12.5	63.5±12.8	68.2±11.1	73.0±14.1
HQ_M[26]	52.0±12.0	66.1±13.8	72.0±12.0	77.1±13.9
PSRC[15]	76.4±12.8	82.3±10.1	88.7±9.3	90.6±7.8
PCRC[36]	74.2±14.2	81.2±11.5	86.1±9.0	90.0±6.4
MSPCRC[36]	81.2±11.7	87.7±9.2	91.6±7.0	93.0±4.8
PMR	78.0±12.2	85.8±10.6	91.1±8.6	93.2±6.6
MSPMR	**84.2±10.7**	**90.2±8.7**	**93.7±7.3**	**96.0±5.3**

### 3. Parameter discussion

**Fig 10:**
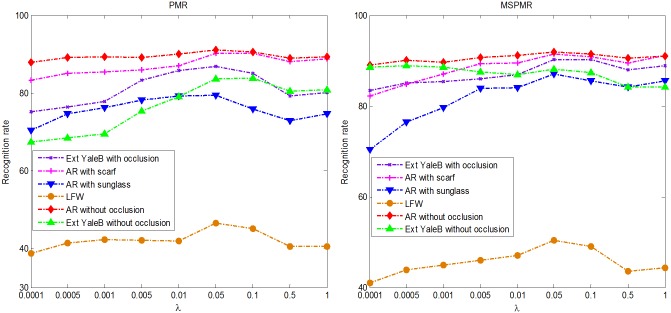
Recognition rate curves of PMR and MSPMR versus the variations in the regularization parameter in different face recognition scenarios.

In this subsection, we mainly discuss how the regularization parameter *λ* affects the performance of our PMR and MSPMR in different face recognition scenarios. The experimental settings are the same as in the aforementioned experiments in section 4.1 and 4.2 except that the training samples per person are fixed at 3. Fig 10 plots the recognition results of PMR and MSPMR versus the variation in the regularization parameter *λ* on different face image databases. We can observe that PMR and MSPMR always achieve their optimal or nearly optimal performance in the range of [0.01, 0.1]. Thus, we can set the regularization parameter of the proposed method in the above range for real-word scenarios.

### 4. Running time comparisons

In this subsection, the CPU runtime of the proposed method is compared with the state-of-the-art methods. The compared results on the AR face database testing with scarves are listed for demonstration. For each individual, 3 samples are randomly chosen for training and another 3 samples for testing. Table 8 tabulates the CPU time cost on all test images conducted using Matlab R2012b on an Intel Core 8 CPU with 3.6 GHz and 8G memory PC at Windows platform. Since the singular value shrinkage operator in matrix regression, the proposed method consumes much more time compared with other methods. Although the patch-based methods have achieved promising results, they come at the cost of expensive running time. Thanks to the independence of the recognition process of each test image, we can hope to save the cost by parallel computation.

**Table 8:** Comparisons of CPU time on AR face database testing with scarves.

Method	Time(s)
CRC[18]	0.6480
SRC[15]	46.3025
NSC[30]	67.9925
HQ_A[26]	108.3425
HQ_M[26]	164.7608
PSRC[15]	71.1593
PCRC[36]	9.5541
MSPCRC[36]	173.5952
PMR	256.9996
MSPMR	4767.1186

### 5. Evaluation of the experimental results

The aforementioned experimental results have shown that the proposed method always obtain better performance than some state-of-the-art methods. However, is this superiority statistically significant? In this subsection, we will assess the experimental results by the null hypothesis statistical test [51]. If the evaluated *p*-value is under the desired significance level (i.e., 0.05), the performance difference between compared approaches is deemed to be statistically significant. The evaluation results are summarized as follows:

1. For face recognition without occlusion, such as on LFW database, MSPMR outperforms MSPCRC significantly for all tests (*p* = 0.014, 0.013,0.016 and 0.020). On other database, although MSPMR performs better than other state-of-the-art methods, the performance discrepancies between MSPMR and other approaches are not statistically significant.
2. For face recognition with occlusion, MSPMR performs significantly better than other approaches in case of real disguise and block occlusion (*p* < 0.001).

## Conclusions and the Future Work

To improve the performance of matrix regression in face of the small sample size problem and preserve the desired performance level in the presence of occlusion and illumination changes, in this paper, we proposed a patch-based matrix regression (PMR) method. PMR first performed matrix regression on each raw patch (without matrix-to-vector conversion), and then combined the recognition outputs of all patches by majority voting. However, it is difficult to pre-define an optimal patch size across different databases. Fortunately, the complementary information across multiple patch scales can be fully utilized to further enhance the recognition performance. To this end, we proposed the multi-scale version of PMR, i.e., MSPMR, to optimally combine the multi-scale outputs. Our extensive experimental results have demonstrated that the proposed methods are more effective and robust than the state-of-the-art methods.

Although our proposed method has obtained successful performance, there are still many issues to be addressed in future. Generally, two main improvements can be made for our method. (1) With the development of the storage device, many images can be collected from real-word applications. One challenge in our method is how to overcome the expensive computational cost. We will try to design efficient matrix regression algorithm to further improve the robustness and effectiveness of our method. (2) In our method, we have to pre-define several specific scale sizes in advance. However, different database may exhibit scale transformation in real-word applications. We can borrow the idea of scale selective local binary patterns [52] to design adaptive scale selection strategy to further improve the flexibility of our method.

### Ethics Statement

Some face image datasets were used in this paper to verify the performance of our methods. These face image datasets are publicly available for face recognition research, and the consent was not needed. The face images and the experimental results are reported in this paper without any commercial purpose.
